# The drug target genes show higher evolutionary conservation than non-target genes

**DOI:** 10.18632/oncotarget.6755

**Published:** 2015-12-24

**Authors:** Wenhua Lv, Yongdeng Xu, Yiying Guo, Ziqi Yu, Guanglong Feng, Panpan Liu, Meiwei Luan, Hongjie Zhu, Guiyou Liu, Mingming Zhang, Hongchao Lv, Lian Duan, Zhenwei Shang, Jin Li, Yongshuai Jiang, Ruijie Zhang

**Affiliations:** ^1^ College of Bioinformatics Science and Technology, Harbin Medical University, Harbin, China; ^2^ Department of Radiology, Second Affiliated Hospital, Harbin Medical University, Harbin, China; ^3^ Genome Analysis Laboratory, Tianjin Institute of Industrial Biotechnology, Chinese Academy of Sciences, Tianjin, China

**Keywords:** drug target, evolutionary conservation, topological properties

## Abstract

Although evidence indicates that drug target genes share some common evolutionary features, there have been few studies analyzing evolutionary features of drug targets from an overall level. Therefore, we conducted an analysis which aimed to investigate the evolutionary characteristics of drug target genes. We compared the evolutionary conservation between human drug target genes and non-target genes by combining both the evolutionary features and network topological properties in human protein-protein interaction network. The evolution rate, conservation score and the percentage of orthologous genes of 21 species were included in our study. Meanwhile, four topological features including the average shortest path length, betweenness centrality, clustering coefficient and degree were considered for comparison analysis. Then we got four results as following: compared with non-drug target genes, 1) drug target genes had lower evolutionary rates; 2) drug target genes had higher conservation scores; 3) drug target genes had higher percentages of orthologous genes and 4) drug target genes had a tighter network structure including higher degrees, betweenness centrality, clustering coefficients and lower average shortest path lengths. These results demonstrate that drug target genes are more evolutionarily conserved than non-drug target genes. We hope that our study will provide valuable information for other researchers who are interested in evolutionary conservation of drug targets.

## INTRODUCTION

Drug targets, a class of biological targets, are *in vivo* binding sites which include receptors, enzymes, ion channels and nucleic acids, etc. Drugs bind to their corresponding targets and perform the desirable therapeutic effects [1]. To date, thousands of drug targets have been identified and stored in databases such as DrugBank [2], Therapeutic Target Database (TTD) [3], Potential Drug Target Database (PDTD) [4] and TDR Targets Database [5].

Previous researches have shown that evolutionary features offer fresh views to many important fields that are related to drug discovery, including immunology [6], physiology [7, 8], epidemiology [9] and neurosciences [10]. Wang et al. [11] conducted an analysis and showed that some targeted genes shared common evolutionary features, which suggested that evolutionary information might provide novel insights for characterizing drug targets from new perspectives.

However, most of the current studies about evolutionary conservation focus on a single gene or several genes belonging to a same protein family, rather than a large group of genes with same or similar features [12–16]. Compared with conventional analyses of evolutionary conservation, gene sets with a large number of genes can better reflect the characteristics of evolution. In addition, evolution conservation can be not only reflected by the general features such as evolutionary rate, the percentage of orthologous genes and protein sequence identity, but also by the network features [17, 18]. Therefore, we wondered whether there was difference in evolutionary features between drug target genes and non-target genes. We hoped to integrate comprehensive evolutionary information and investigate the evolutionary conservation characteristics of drug target genes from a global perspective.

Therefore, we compared the evolutionary features between drug target genes and non-target genes combining both regular evolutionary features and some network features. All the evolutionary features were categorized into two groups: (1) evolutionary features of 21 species including evolutionary rate, conservation score and the percentage of orthologous genes; (2) topological features of human protein-protein interaction network including the average shortest path length, betweenness centrality, clustering coefficient and degree. In this research, we hope to explore the evolutionary conservation features of drug targets and help to enhance the efficiency of target identification.

## RESULTS

### Drug target genes had lower evolutionary rates than non-target genes

**Figure 1:**
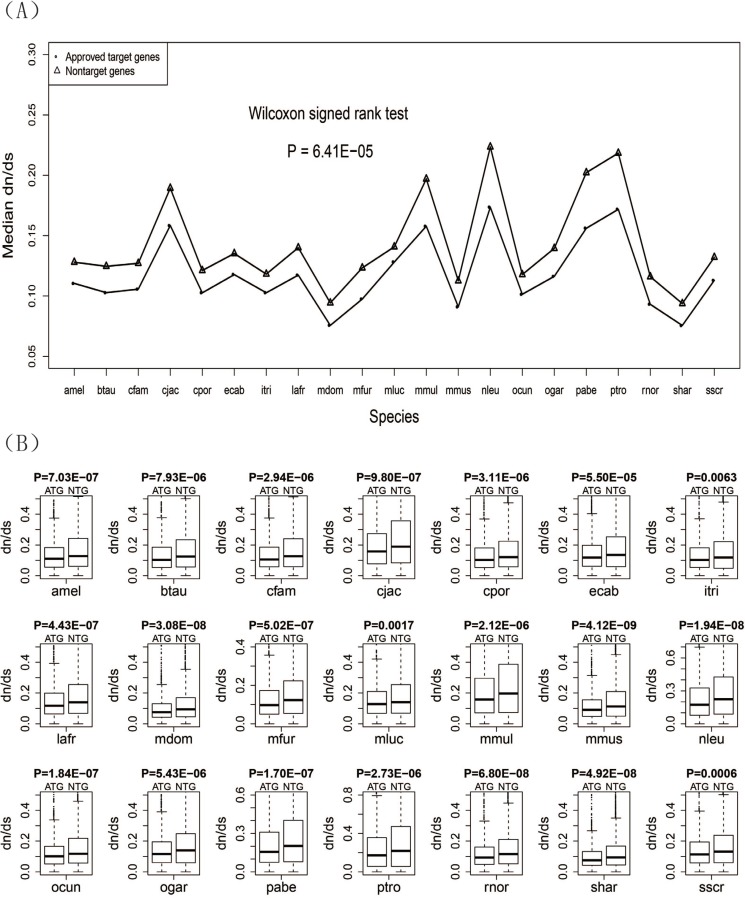
Evolutionary rates (dN/dS ratios) for the drug target genes and non-target genes (**A**) Line chart of the drug target genes and non-target genes. (**B**) Box plots of the drug target genes against non-target genes for each of the 21 species.

For each of the 21 species, we calculated the evolutionary rate dN/dS of both the drug target genes and non-target genes. We also respectively calculated the median dN/dS of drug target genes and non-target genes for each species and compared them using a line chart (Figure 1A). The results showed that the median dN/dS of drug target genes was significantly lower than that of non-target genes (*P* = 6.41E−05). For each species, a box plot was given to display the difference of dN/dS between the two groups of genes (Figure 1B). The results of box plots and Wilcoxon rank sum tests showed that the evolutionary rate of drug target genes was lower than that of the non-drug target genes for each of the 21 species. Detailed information about the dN/dS for each species is given in Table 1.

**Table 1:** Summary statistics for the comparisons of dn/ds in species

Species	dn/ds of Approved Drug Target Genes			dn/ds of Non-Target Genes			Wilcoxon Rank Sum test *P*-value
	Median	Upper Quartile	Lower Quartile	Median	Upper Quartile	Lower Quartile	
amel	0.1104	0.1831	0.0555	0.1280	0.2426	0.0608	7.03E–07
btau	0.1028	0.1851	0.0535	0.1246	0.2344	0.0564	7.93E–06
cfam	0.1057	0.1857	0.0576	0.1270	0.2408	0.0591	2.94E–06
cjac	0.1584	0.2733	0.0779	0.1893	0.3575	0.0838	9.80E–07
cpor	0.1026	0.1800	0.0534	0.1211	0.2247	0.0578	3.11E–06
ecab	0.1177	0.1984	0.0613	0.1352	0.2528	0.0595	5.50E–05
itri	0.1027	0.1817	0.0538	0.1181	0.2212	0.0487	0.0063
lafr	0.1173	0.1990	0.0645	0.1400	0.2551	0.0684	4.43E–07
mdom	0.0757	0.1308	0.0425	0.0943	0.1692	0.0451	3.08E–08
mfur	0.0975	0.1736	0.0502	0.1233	0.2235	0.0537	5.02E–07
mluc	0.1281	0.2104	0.0684	0.1407	0.2547	0.0693	0.00172
mmul	0.1578	0.2966	0.0709	0.1970	0.3870	0.0730	2.12E–06
mmus	0.0910	0.1558	0.0479	0.1125	0.2100	0.0497	4.12E–09
nleu	0.1735	0.3260	0.0781	0.2235	0.4261	0.0881	1.94E–08
ocun	0.1014	0.1662	0.0510	0.1178	0.2184	0.0570	1.84E–07
ogar	0.1163	0.1950	0.0604	0.1395	0.2482	0.0593	5.43E–06
pabe	0.1561	0.3096	0.0743	0.2022	0.4018	0.0792	1.70E–07
ptro	0.1718	0.3559	0.0578	0.2184	0.4715	0.0574	2.73E–06
rnor	0.0931	0.1616	0.0487	0.1159	0.2105	0.0521	6.80E–08
shar	0.0756	0.1326	0.0426	0.0938	0.1676	0.0451	4.92E–08
sscr	0.1130	0.1944	0.0585	0.1321	0.2378	0.0595	0.0006

### Drug target genes had higher conservation scores than non-target genes

**Figure 2:**
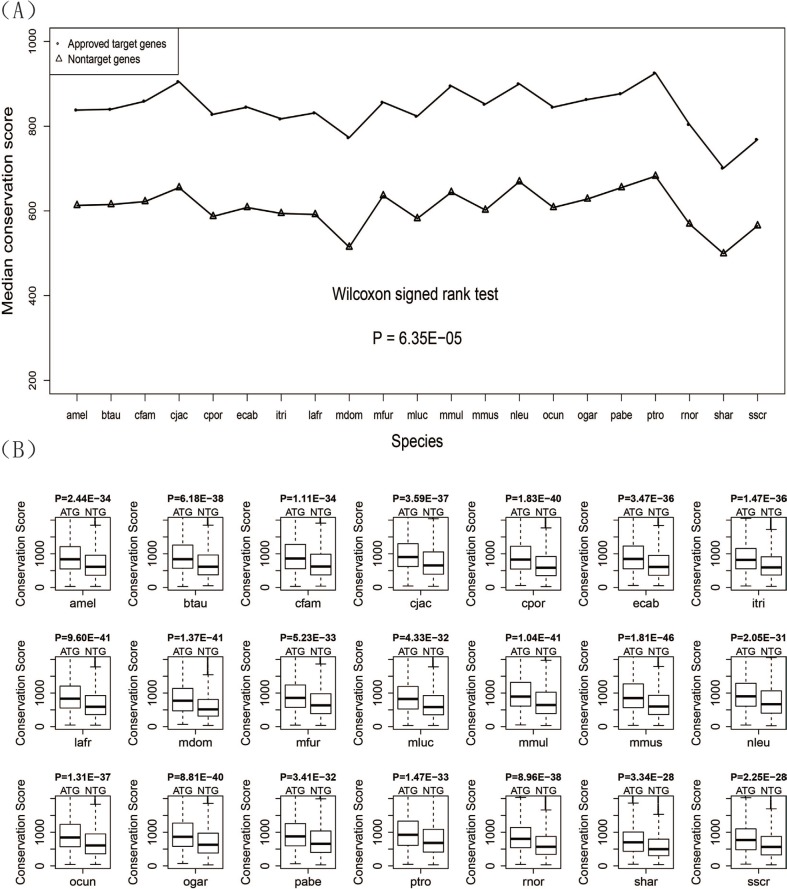
Conservation scores for the drug target genes and non-target genes (**A**) Line chart of the drug target genes and non-target genes. (**B**) Box plots of the drug target genes against non-target genes for each of the 21 species.

We aligned the protein sequence of both human drug target genes and non-target genes to the orthologous protein sequence of the other 21 species by using BLAST software and got conservation scores from the blast results. The median conservation scores of the two gene sets for 21 species were calculated and displayed by a line chart (Figure 2A) showing that the median conservation score of drug target genes was higher than that of non-target genes. The Wilcoxon signed rank test gave a *P*-value of 6.40E-05 confirming that there was significant difference in the conservation scores between human drug target genes and non-target genes. For each of the 21 species, the conservation scores of drug target genes are significantly higher than that of the non-target genes (Figure 2B). Detailed information about the conservation score for each species is given in Table 2.

**Table 2:** Summary statistics for the comparisons of conservation score in species

Species	Sequence Identity of Approved drug Target Genes			Sequence Identity of Non-Target Genes			Wilcoxon Rank Sum test *P*-value
	Median	Upper Quartile	Lower Quartile	Median	Upper Quartile	Lower Quartile	
amel	838.00	1213.00	548.00	613.00	957.00	361.00	2.44E–34
btau	840.00	1257.50	571.50	615.00	965.00	373.50	6.18E–38
cfam	859.00	1279.00	557.25	622.00	988.00	371.00	1.11E–34
cjac	905.00	1299.50	620.00	655.00	1054.25	394.00	3.59E–37
cpor	828.00	1221.00	545.00	587.00	919.50	352.00	1.83E–40
ecab	845.00	1228.00	552.50	608.00	952.00	360.25	3.47E–36
itri	817.50	1153.00	553.25	594.00	909.00	367.50	1.47E–36
lafr	831.50	1205.25	555.00	591.50	926.00	359.00	9.60E–41
mdom	773.00	1135.75	472.00	514.50	808.25	314.00	1.37E–41
mfur	856.50	1238.75	576.00	636.00	981.25	389.00	5.23E–33
mluc	823.50	1197.00	525.00	582.00	925.00	354.00	4.33E–32
mmul	895.00	1315.75	613.00	644.00	1023.50	390.00	1.04E–41
mmus	852.00	1271.50	565.00	602.00	932.00	361.00	1.81E–46
nleu	900.00	1290.50	610.00	669.00	1064.00	403.00	2.05E–31
ocun	845.00	1233.25	568.50	608.00	949.75	360.00	1.31E–37
ogar	863.00	1272.25	580.00	628.00	974.00	382.00	8.81E–40
pabe	877.00	1257.50	595.50	655.00	1038.00	399.00	3.41E–32
ptro	925.50	1332.00	611.00	682.00	1087.00	410.00	1.47E–33
rnor	804.00	1141.00	541.50	569.00	876.00	343.00	8.96E–38
shar	701.00	1007.00	432.50	499.00	796.00	305.00	3.34E–28
sscr	768.50	1098.75	482.25	565.00	876.00	328.00	2.25E–28

### Drug target genes had higher percentages of orthologous genes than non-target genes

**Figure 3:**
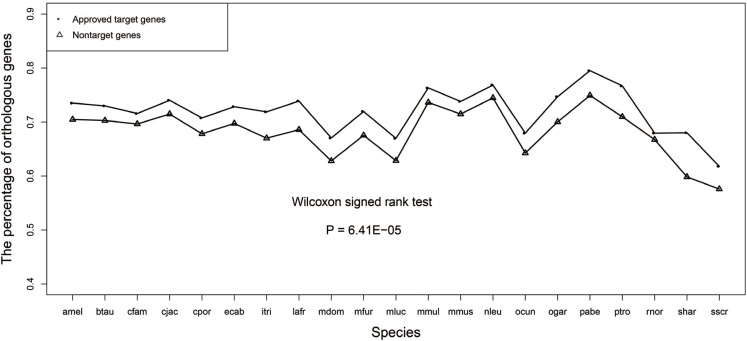
Line chart of the percentage of orthologous genes for the drug target genes and non-target genes

We calculated the percentage of orthologous genes of drug target genes and non-target genes for each species and displayed the line chart of this evolutionary feature in Figure 3, which showed that the drug target genes had a higher percentage of orthologous genes than the non-target genes. The *P*-value of Wilcoxon signed rank test was 9.54E-07 confirming that there was significant difference in the percentage of orthologous genes between the two groups of genes.

### Drug target genes had a tighter network topology structure than non-target genes

**Figure 4:**
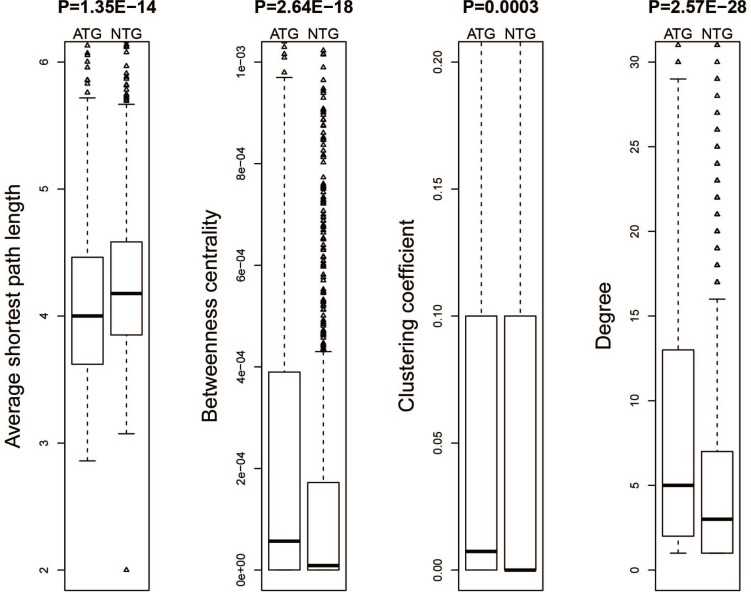
Network topological properties for the drug target genes and non-target genes

We further analyzed the topological properties of the human protein-protein interaction network downloaded from HPRD and extracted the network features of both drug target genes and non-target genes. Then we compared these features between drug target genes and non-target genes. These following results were obtained: 1) The average shortest path length of drug target genes was significantly smaller than that of non-target genes (Figure 4A) and 2) The betweenness centrality, clustering coefficient and degree of drug target genes were significantly higher than those of non-target genes (Figure 4B–4D). These results showed that drug target genes had a tighter topological structure than non-target genes in the human protein-protein interaction network.

## DISCUSSION

It is an important task to investigate the evolutionary conservation of drug target genes, which helps to well characterize drug targets. In this study we analyzed the evolutionary conservation of drug target genes by comparing multiple evolutionary characteristics including both classical features (evolutionary rate, conservation score and percentage of orthologous genes) and network topological properties (average shortest path length, betweenness centrality, clustering coefficient and degree). Through comprehensive analyses, we got consistent results supporting that drug target genes were more evolutionarily conserved during the evolutionary history.

Previous studies about drug targets have identified genes or genome regions with higher evolutionary conservation as potential or candidate drug targets. For instance, the nucleoprotein (NP) of the influenza A virus which is a protein of high conservation was identified as a potential target of universally effective antivirals [19]. Heat shock proteins (HSPs), a ubiquitous group of evolutionary conserved proteins, which are involved in binding antigens and presenting them to the immune system, were determined as possible therapeutic targets [20]. Nonstructural proteins (NS3) are components of flavivirus polyprotein. Shiryaev et al. [21] performed a study focusing on the structural and functional characteristics of flaviviral protease. They found that the N-terminal and C-terminal parts of NS3 were composed by serine protease and the RNA helicase. Individual virus proteins were produced and a new progeny would be assembled if the polyprotein was cleaved by protease or RNA helicase. Since both the protease and the RNA helicase were conserved among flaviviruses, NS3 was identified as a promising drug target in flaviviral infections.

Furthermore, some genes or proteins involved in conserved cellular progress such as DNA replication and apoptosis during evolution can also be identified as potential drug targets. For instance, Robinson et al. [22] explored the architecture and conservation of the bacterial DNA replication machinery and found that genes or proteins involved in maintaining the machinery of DNA replication had the greatest potential as drug targets. The mitochondrial permeability transition (mPT) is a mechanism that enables the secretion of Cytochrome-c (Cyt-c), Apoptosis Inducing Factor (AIF) and other pro-apoptotic proteins which initiate and promote apoptosis. A research conducted by Hellebrand et al. [23] suggested that some mPT inhibitory agents might become promising drug targets against apoptosis.

With the rapid development of computer technology and machine learning theory, evolution information has been used to identify and prioritize drug targets. Ludin et al. [24] predicted antimalarial drug target candidates by utilizing evolution information and found 40 candidate drug targets with high evolution conservation. Another study about drug target identification and prioritization also indicated that many potential drug target genes could be predicted by orthologues information [25].

The comparison analysis results obtained in our study and the previous studies focusing on evolutionary conservation of drug targets or drug target identification based on evolution information suggest that drug targets are closely correlated with evolution conservation and they are characterized by higher evolutionary conservation during evolution process compared with non-target genes. This indicates that the results in our study are quite reliable and they might have the potential to expand the understanding of evolutionary characteristics of drug target genes.

## MATERIALS AND METHODS

### Human drug target genes

The human drug target gene set used in our study came from the DrugBank database that is a unique bioinformatics and cheminformatics resource combing detailed drug data with comprehensive drug target information [2]. We downloaded the data of Food and Drug Administration (FDA) approved drugs and the corresponding drug targets, which contained a total of 1857 terms for multiple species. We then extracted the human drug targets from the original data and finally obtained 1347 FDA-approved drug target genes for the following analyses.

### Non-target genes

With the purpose of getting non-target gene set, we downloaded protein family data from Pfam database (ftp://ftp.sanger.ac.uk/pub/databases/Pfam/releases/Pfam27.0/), a collection of protein families, each represented by multiple sequence alignments and hidden Markov models (HMMs) [26], and obtained the human protein family information. After filtering out the protein families to which drug targets belonged, we got the non-target gene set containing 4181 non-redundant genes. It's worth noting that non-targets refer to those proteins that do not have similar domains with target proteins.

### Calculation of evolutionary rate, percentage of orthologous genes and conservation score

We downloaded the orthologous gene data which included 21 species from the Ensembl database [27–29] (ftp://ftp.ensembl.org/pub/release-69/mysql/ensembl_mart_69). The full names and abbreviations of the 21 species can be found in Table 3. Then we extracted one-to-one ortholog genes [30] with non-null dN (rate of non-synonymous substitutions) and dS (rate of synonymous substitutions) values and calculated the evolutionary rate as the ratio of dN/dS.

**Table 3:** Full names and abbreviations

Calss	Abbreviation	Full name
Species 1	amel	Ailuropoda melanoleuca
Species 2	btau	Bos taurus
Species 3	cfam	Canis familiaris
Species 4	cjac	Callithrix jacchus
Species 5	cpor	Cavia porcellus
Species 6	ecab	Equus caballus
Species 7	itri	Ictidomys tridecemlineatus
Species 8	lafr	Loxodonta africana
Species 9	mdom	Monodelphis domestica
Species 10	mfur	Mustela putorius furo
Species 11	mluc	Myotis lucifugus
Species 12	mmul	Macaca mulatta
Species 13	mmus	Mus musculus
Species 14	nleu	Nomascus leucogenys
Species 15	ocun	Oryctolagus cuniculus
Species 16	ogar	Otolemur garnrttii
Species 17	pabe	Pongo abelii
Species 18	ptro	Pan troglodytes
Species 19	rnor	Rattus norvegicus
Species 20	shar	Sarcophilus harrisii
Species 21	sscr	Sus scrofa

For both drug target genes and non-target genes, we counted the numbers of one-to-one orthologous genes in each of the 21 species and then calculated the percentage of orthologsous genes for each species.

Conservation score is defined as a score assigned to each orthologous gene by sequence alignment between species to determine how conserved a gene is. Here the sequence conservation score is used to evaluate the degree of similarity between a human sequence and another species sequence for the orthologous gene. The higher scores indicate the higher degree of conservation. To compute the sequence conservation score, we downloaded the pair-wise protein sequences of human and other species from BioMart [31] (http://www.ensembl.org/biomart/martview) and performed alignment using BLASTP program and the BLOSUM62 matrix [32].

### Calculation of topological properties of human protein-protein interaction network

We downloaded the protein-protein interaction (PPI) network data containing 39240 interaction pairs from the Human Protein Reference Database (HPRD) [33]. In the PPI network, a node denotes a protein and a path denotes a finite sequence of edges which connect proteins. Then we calculated 4 topological properties which included the average shortest path length, betweenness centrality, clustering coefficient and degree [34] by using MCODE, a plug-in of Cytoscape software [35]. The average shortest path length reflecting how tight one node is connected to the other nodes in a network is defined as the average length of all shortest paths passing through a certain node. The normalized betweenness centrality of node *v* is defined as $Bv=\frac{1}{\left(n-1)(n-2\right)}\sum_{i\neq j\neq v\in V}\frac{\sigma \text{ivj}}{\sigma ij}$ where σ_*ij*_ is the number of shortest paths from node *i* to node *j* and is the number of shortest paths passing through node *v* out of σ_ivj_. The betweenness centrality is an indicator used to measure a node's centrality in a network. The clustering coefficient in an undirected network is defined as $\text{CCv}=\frac{n}{C_{k}^{2}}=\frac{2n}{k(k-1)}$ where *n* is the number of edges connecting the *k* direct neighbors of node *v* and *C*_k_^2^ is the max possible number of edges between *k* nodes. The clustering coefficient represents the degree to which nodes in a network tend to cluster together. The degree of node *v* is the number of nodes directly connecting with node *v*. To compare the network features of the drug target genes and non-target genes, we extracted the topological properties for the two gene sets.

### Statistical analysis

We used the Wilcoxon rank sum test to evaluate the statistical significance of the difference in an evolutionary feature or a network feature between the drug target genes and non-target genes. We used the Wilcoxon signed rank test to check whether the median of an evolutionary feature of drug target genes was significantly different from that of the non-target genes for each species. In our study, Perl scripts were used for data processing (http://www.activestate.com/activeperl) and R scripts were used for statistical graphics and calculations (http://cran.r-project.org).
